# Effect of aspirin use on survival benefits of breast cancer patients: A meta-analysis

**DOI:** 10.1097/MD.0000000000026870

**Published:** 2021-08-20

**Authors:** Jiamin Liu, Fengxian Zheng, Meng Yang, Xiaoyong Wu, Aimin Liu

**Affiliations:** a College of Medicine, Guangxi University of Traditional Chinese Medicine, Nanning, China; b Department of Critical Care Medicine, Affiliated Danzhou People's Hospital of Hainan Medical University, Hainan, China; c Department of Dermatology, The Third Affiliated Hospital of Guangxi Medical University, Nanning, Guangxi, China; d Department of General Surgery, Affiliated Danzhou People's Hospital of Hainan Medical University, Hainan, China; e Department of Basic Nursing, School of Nursing, Kunming Medical University, Kunming, China.

**Keywords:** aspirin, breast cancer, meta-analysis, survival

## Abstract

**Objective:**

The purpose of this study is to investigate whether aspirin improves the prognosis of breast cancer patients by meta analysis.

**Methods:**

Searched PubMed, EMBASE, and other databases for literature on the relationship between aspirin use and breast cancer prognosis, with the deadline of October 2019. The related results of all-cause death, breast cancer-specific death, and breast cancer recurrence/metastasis were extracted to combine the effect amount. The sensitivity analysis and published bias analysis were carried out for the included data. Stata12.0 software was used to complete all statistical analysis.

**Results:**

A total of 13 papers were included in the study, including 142,644 breast cancer patients. The results of meta-analysis showed that patients who took aspirin were associated with lower breast cancer-specific death (HR = 0.69, 95% CI = 0.61–0.76), all-cause death (HR = 0.78, 95% CI = 0.71–0.84), and risk of recurrence/metastasis (HR = 0.91, 95% CI: 0.82–1.00).

**Conclusions:**

Aspirin use may improve all-cause mortality, specific mortality, and risk of recurrence/metastasis in patients with breast cancer.

## 1 Introduction

Breast cancer (BC) is a common malignant tumor in women worldwide, affecting approximately 12% of women.^[1]^ According to research by Ferlay et al,^[2]^ about 2088.8 million women were diagnosed with breast cancer, which is the most common cancer among women in the world except South Africa, and more than 500,000 people die of breast cancer every year.^[3]^ In addition, in the study of Wu et al,^[4]^ the metastatic rate of breast cancer was 7.7%. According to reports, 5% to 10% of breast cancers were caused by genes, and 90% to 95% of the environment was determined^[5]^ such as: use of hormonal drugs, environmental deterioration, unhealthy behavioral lifestyles, mental and psychological factors, etc.^[6–8]^ Therefore, in the past few years, nonsteroidal anti-inflammatory drugs (NASIDs) have been applied to the treatment and prevention of breast cancer, including ibuprofen, nimesulide, celecoxib, aspirin, etc.

Aspirin has antipyretic, analgesic, and anti-inflammatory effects and the preventive effect of aspirin on colon cancer, breast cancer, and gastric cancer has also been confirmed^[9,10]^ and several studies have found that the use of aspirin can reduce breast cancer mortality,^[11]^ however, another part of the study proves that taking aspirin can increase breast cancer mortality.^[12]^ Sharpe et al^[13]^ found that aspirin can reduce the risk of breast cancer recurrence, in contrast to the results of Bens et al.^[14]^ Therefore, the results of aspirin on reducing breast cancer mortality and breast cancer recurrence and metastasis are still controversial.

In this light, we conducted a meta-analysis of the relationship between oral aspirin and specific death, all-cause death, and recurrence in patients with breast cancer. We also hope that our work can provide evidence-based medical evidence for the treatment of breast cancer.

## 2 Method

### 2.1 Publication search

We searched the papers in the PubMed, Embase, and other databases. The search time was limited to the establishment of the database until October 30, 2019. The following keywords were retrieved: “aspirin,” “nonsteroidal anti-inflammatory drugs,” “NSAIDs,” “breast cancer.” This search was completed independently by 2 investigators.

### 2.2 Inclusion and exclusion of publications

Inclusion: The outcomes of the papers included breast cancer-specific death, all-cause death, and breast cancer recurrence/metastasis. The studied population intake aspirin. HR with 95% CI was used to evaluate the data. Cohort study.

Exclusion: HR with 95% CI was not used to evaluate data. The object being studied is not human. Study type is not a cohort study.

### 2.3 Data extraction

The following data information was extracted from all selected studies: first author, year of publication, the city where the participants are located, type of study, follow-up time, number of samples, number of people taking/not taking aspirin, outcome, *HR* with *95% CI*. The 2 researchers cross-checked the results of the papers, and the differences that occurred during the screening process were not discussed, the third party involved in the discussion and decision.

### 2.4 Quality evaluation

The Newcastle-Ottawa Scale^[15]^ was used by 2 researchers to evaluate the quality of the selected papers, and the evaluation process was completed independently. When the results of 2 independent investigators were different, the discussion would be held first, when the opinions were still inconsistent, the third investigator would conduct the quality evaluation.

### 2.5 Ethical statements

No ethical approval is required since this is a literature-based study.

### 2.6 Statistical analysis

The aggregated statistical data from the meta-analysis was HR and 95% CI. Statistical heterogeneity between studies was performed using the Q test and the value of *I*^*2*^ was calculated. If *P* ≥ .1 and *I*^*2*^ ≤ 50%, there was no statistical heterogeneity between the studies, and the combined effect model was used for the combined analysis. On the contrary, *P* < .1 and *I*^*2*^ > 50%, there was statistical heterogeneity between the studies, and the random effects model was used for the combined analysis. Sensitivity analysis was used to evaluate the impact of each study on the overall hazard ratio. Egger test was used to assess the trial error. STATA version 12.0 was used for us to analysis the data. Significance test level was *α* = 0.05.

## 3 Results

### 3.1 Basic characteristics and quality evaluation of the research

**Figure 1:**
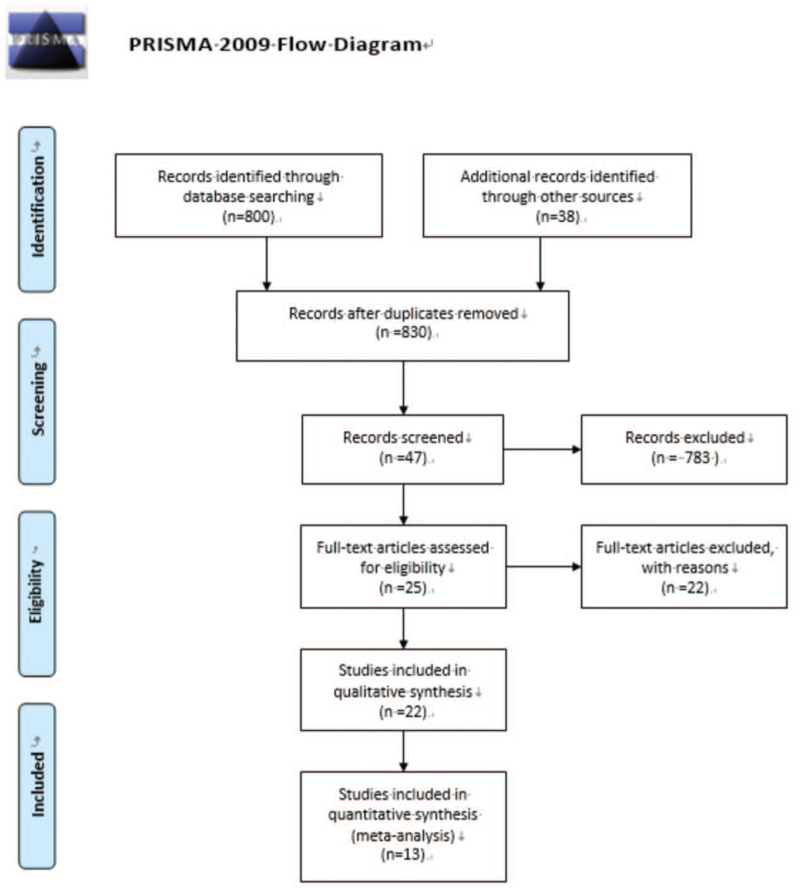
Retrieving flow chart.

Initially, a total of 838 papers were retrieved, 590 from PubMed, 248 from Embase. After reading the abstract and the title, there were 55 papers remaining. Finally, a total of 13^[11,16–27]^ studies were included to read the full text. A total of 142,644 research samples were included. The search process was shown in Figure 1.

Table 1 summarized the basic characteristics of the included studies. In all papers, 6 were conducted in America,^[16–18,21–22,24]^ 2 were conducted in United Kingdom,^[11,23]^ 2 were organized in Denmark,^[26,27]^ 2 were organized in Ireland,^[19,20]^ and other was launched in Sweden.^[25]^ A total of 142,644 participants were included. Outcome events in 11 studies were breast cancer-specific deaths,^[11,16–25]^ 10 studies included all-cause death outcome events,^[11,16–24]^ and 4 studies reported recurrence/metastasis rates.^[22,25–27]^ Document quality evaluation was showed in Table 2.

**Table 1:** Basic characteristics of studies.

Author	Year of publication	Origin	Type of study	Follow-up time	Number of samples (n)	Use aspirin/no use (n)	Outcome	Breast cancer-specific death	All-cause death	Recurrence/metastasis
Blair CK^[16]^	2007	America	Cohort study	1992–2001	591	254/337	1,2	0.53 (0.30,0.93)	0.53 (0.36,0.79)	
Wernli KJ^[17]^	2011	America	Cohort study	1998–2006	3022	1059/1963	1,2	0.64 (0.37,1.37)	0.91 (0.65,1.29)	
Li Y^[18]^	2012	America	Cohort study	1996–2006	1024	Not clear	1,2	0.89 (0.53,.52)	0.82 (0.54,1.24)	
Fraser DM^[11]^	2014	England	Cohort study	1998–2008	2617	815/1802	1,2	0.42 (0.31,0.55)	0.53 (0.45,0.63)	
Barron TI^[19]^	2014	Ireland	Cohort study	2000–2006	2796	740/2056	1,2	0.99 (0.68, 1.45)	1.11 (0.83, 1.50)	
Barron TI^[20]^	2015	Ireland	Cohort study	2001–2012	4540	764/3776	1,2	0.98 (0.74,1.30)	1.10 (0.90,1.33)	
Bradley MC^[21]^	2016	America	Cohort study	1993–2009	2925	1274/1651	1,2	0.95 (0.68,1.31)	0.93 0.75,1.15	
Shiao J^[22]^	2016	America	Cohort study	1998–2016	222	65/157	1,2,3	0.41 (0.20,0.83)	0.67 (0.35,1.27)	0.34 (0.15,0.81)
Mc Menamin MC^[23]^	2017	England	Cohort study	2009–2015	15,140	2822/12,318	1,2	0.92 (0.75,1.14)	1.21 (1.04,1.40)	
Wang T^[24]^	2019	America	Cohort study	1996–2014	1442	301/1141	1,2	0.87 (0.59,1.29)	1.21 (0.99,1.48)	
Frisk G^[25]^	2018	Sweden	Cohort study	2006–2012	21,414	9582/11,832	1,3	0.99 (0.79, 1.23)		0.97 (0.86,1.10).
Cronin-Fenton DP^[26]^	2016	Denmark	Cohort study	1996–2008	34,188	6802/27,386	3			1.0 (0.85, 1.3)
Bens A^[27]^	2018	Denmark	Cohort study	1996–2012	52,723	5295/47,428	3			0.88 (0.69,1.13)

Note: 1: Breast cancer-specific death; 2: all-cause death; 3: recurrence (metastasis).

**Table 2:** Quality evaluation of studies.

	Selection				Comparability	Outcome			
	Representativeness of the exposed cohort	Selection of nonexposure group	Exposure confirmation	Outcome events before the start of the study	Comparability between research design and structure	assessment of outcome	was follow-up long enough for outcomes to occur	adequacy of follow-up of cohort	Total
Blair CK^[16]^	0	1	0	1	2	1	1	1	7
Wernli KJ^[17]^	0	1	0	1	2	1	1	1	7
Li Y^[18]^	0	1	0	1	2	1	1	0	7
Fraser DM^[11]^	0	1	1	1	2	1	1	1	8
Barron TI^[19]^	0	1	1	1	2	1	1	0	7
Barron TI^[20]^	0	1	1	1	2	1	1	1	8
Bradley MC^[21]^	0	1	0	1	2	1	1	1	7
Shiao J^[22]^	0	1	1	1	0	1	1	1	6
Mc Menamin MC^[23]^	0	1	1	1	2	1	1	0	7
Wang T^[24]^	0	1	0	1	2	1	1	0	6
Frisk G^[25]^	0	1	1	1	2	1	1	1	8
Cronin-Fenton DP^[26]^	0	1	1	1	2	1	1	0	7
Bens A^[27]^	0	1	1	1	2	1	1	1	8

### 3.2 Breast cancer-specific death

**Figure 2:**
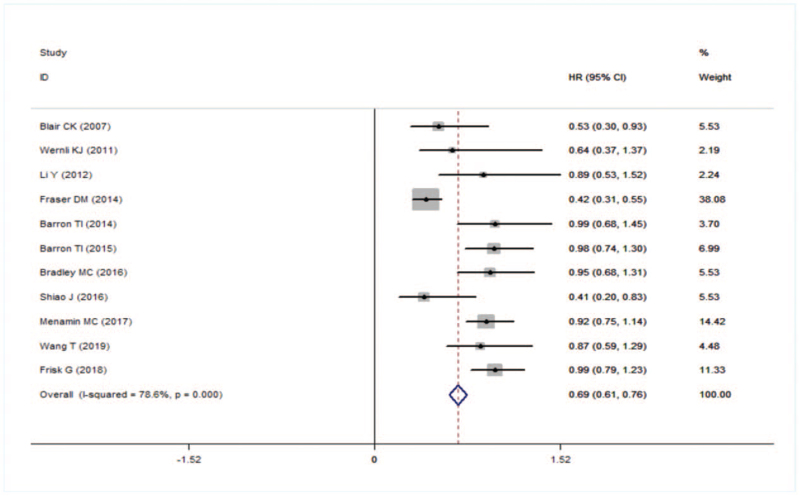
Forest plot of specific death.

In all studies included in the meta-analysis, the use of aspirin reduced the specific death of breast cancer (HR = 0.69, 95% CI: 0.61–0.76). Higher heterogeneity between studies (*I*^*2*^ = 78.6%, *P* = .000) (Fig. 2).

**Figure 3:**
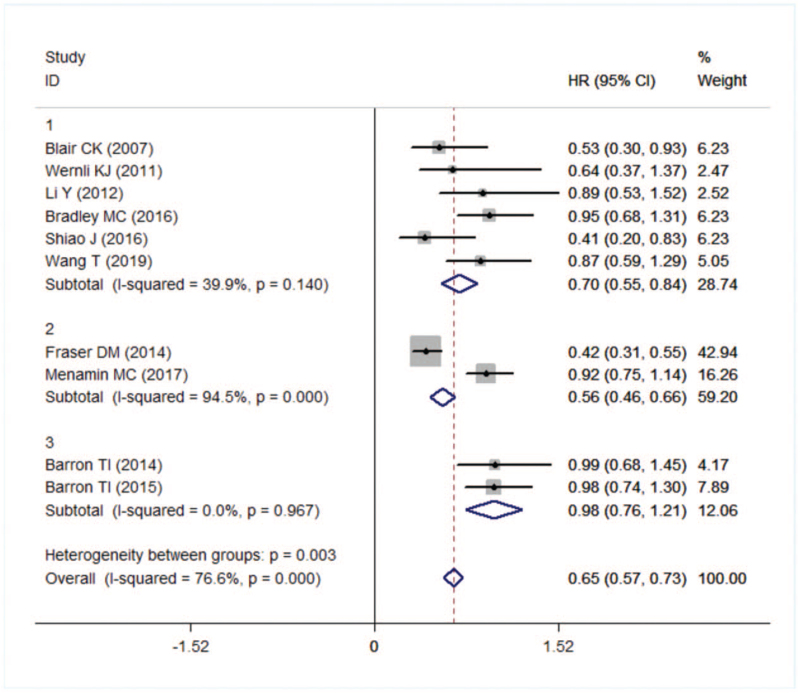
Forest plot of specific death in different country.

Then we conducted a subgroup analysis based on the source of the city (1 = United States, 2 = UK, 3 = Ireland). In addition, the use of aspirin in the United States and the United Kingdom reduced the risk of breast cancer-specific death by 30% and 44%, respectively (HR = 0.70, 95% CI: 0.55–0.84; HR = 0.56, 95% CI: 0.46–0.66), while the use of aspirin in Ireland was not associated with breast cancer-specific death (HR = 0.98, 95% CI: 0.76–1.21). However, heterogeneity was not found by subgroup analysis (Fig. 3).

**Figure 4:**
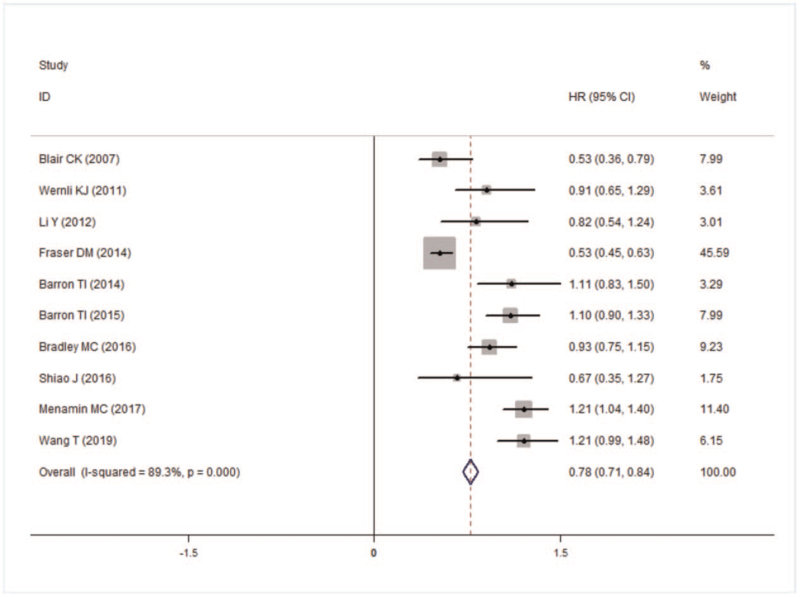
Forest plot of all-cause death.

Sensitivity analysis showed that the study of Fraster^[11]^ was an outlier, and the reconsolidation analysis was excluded (Fig. 4). The meta-analysis of 10 studies showed that the aspirin-specific death was 15% lower than the no used (HR = 0.85, 95% CI: 0.76–0.96), and the heterogeneity result was (*I*^*2*^ = 43.6%, *P* = .068). Egger’ test results suggested that there was no publication bias (*t* = 1.22, *P* = .255).

### 3.3 All-cause death

The results showed that taking aspirin could reduce the risk of all-cause death in breast cancer patients (HR = 0.78, 95% CI: 0.71–0.84). However, there was heterogeneity between the papers (*I*^*2*^ = 0.83, *P* = .000) (Fig. 4).

**Figure 5:**
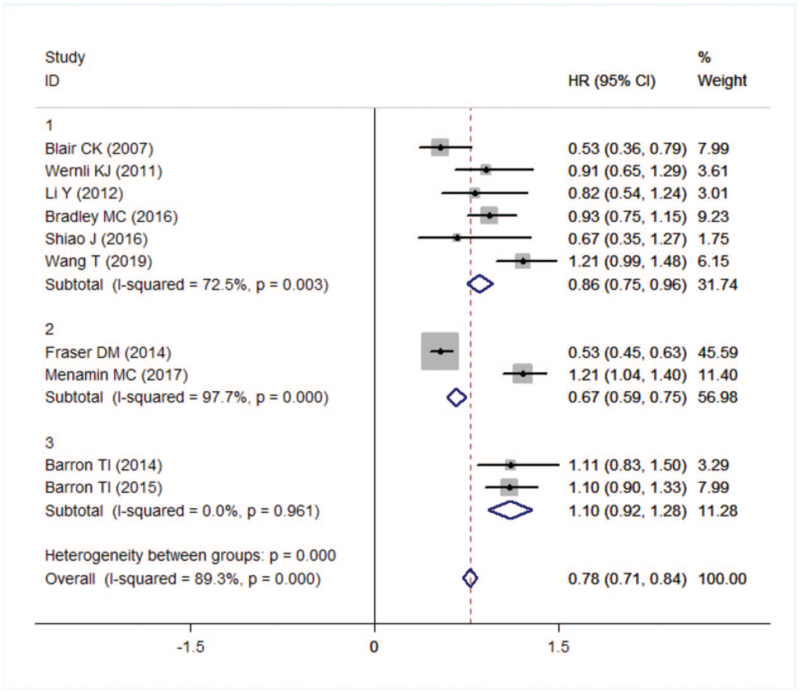
Forest plot of all-cause death in different country.

A subgroup analysis based on national sources revealed no statistically significant studies from Ireland (HR = 1.10, 95% CI: 0.92–1.28) while reduced all-cause mortality from studies in the United States (HR = 0.86, 95% CI: 0.75–0.96) and the United Kingdom (HR = 0.67, 95% CI: 0.59–0.75) (Fig. 5). However, heterogeneity sources were not found in subgroup analyses.

The sensitivity analysis showed that all-cause death of Fraser^[11]^ and Mc Menamin^[23]^ studies were outliers. After remerging, the heterogeneity changes little, indicating that the results were stable. Egger test results suggested that there was no publication bias (*t* = 0.57, *P* = .582).

### 3.4 Recurrence/metastasis

**Figure 6:**
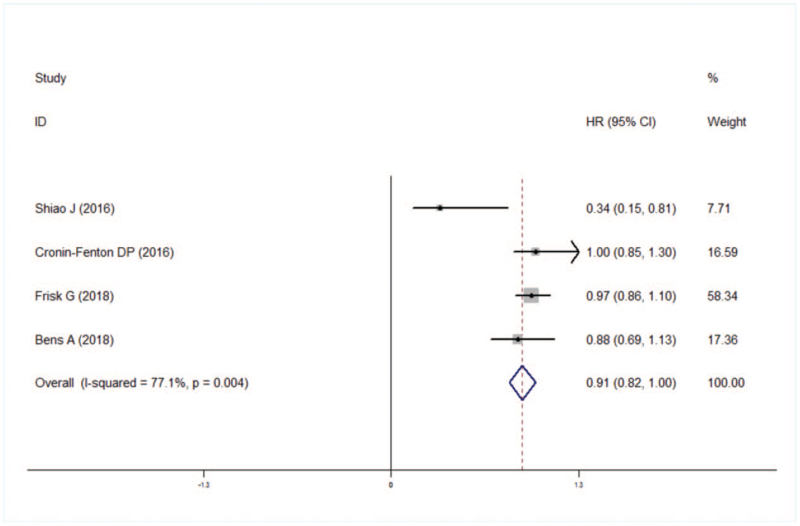
Forest plot of recurrence/metastasis.

Meta-analysis showed that risk of breast cancer patients using aspirin was associated with lower risk recurrence/metastasis (HR: 0.91, 95% CI: 0.82–1.00), but there was heterogeneity between studies (*I*^*2*^ = 77.1%, *P* = .004) (Fig. 6).

Sensitivity analysis did not reveal the source of heterogeneity. Egger test results suggested that there was no publication bias (*t* = 2.58, *P* = .123).

## 4 Discussion

A total of 13^[11,16–27]^ studies were selected for this meta-analysis, and they included 140,644 breast cancer patients. Our meta-analysis found that the use of aspirin reduced breast cancer-specific death by 31% and all-cause death by 22%, while risk of recurrence/metastasis by 9%. So the use of aspirin was beneficial for breast cancer patients.

Aspirin is a nonsteroidal anti-inflammatory drug that is mainly used for antipyretic and analgesic, anti-inflammatory, anti-rheumatic, and anti-platelet aggregation. In recent years, more and more studies have shown that aspirin can reduce the risk of breast cancer death and recurrence.^[28]^ The mechanism of action of aspirin on breast cancer has also been proved by some experiments. Recent reports by Hsieh and Wang^[29]^ demonstrate that aspirin inhibits crosstalk between 4T1 and RAW 264.7 cells and regulates M1/M2 macrophage subtypes by regulating angiogenesis and inflammatory mediator production, thereby contributing to the treatment of breast cancer. Another study found that the anti-tumor effect of aspirin mainly inhibits the activity of cyclooxygenase in the body, thereby inhibiting breast cancer cell proliferation, tumor angiogenesis, and tumor cell infiltration^[30]^ Another mechanism of action of aspirin on breast cancer is by anti-adenocarcinoma by inhibiting NF-κB and TGF-β/SMAD-mediated signaling pathways.^[31]^

Subgroup analysis by country source showed that the use of aspirin reduced the risk of specific death and all-cause mortality in both the United States and the United Kingdom, while that the use of aspirin was not associated with all-cause death and specific death in breast cancer patients, in Ireland. May be due to different lifestyles and eating habits in different countries, resulting in different prognosis of breast cancer.^[32]^ A subgroup analysis of breast cancer-specific deaths found no heterogeneity between the 2 subgroups of the United States and Ireland, but the subgroup of heterogeneities in the 2 UK studies included. The possible causes of heterogeneity are that the sample size of Mc Menamin et al^[23]^ is much higher than that of Fraser et al, and another reason may be that the follow-up time of the 2 studies is different, Mc Menamin et al^[23]^ The study was followed for 6 years, and the cohort of Fraser et al^[11]^ was followed for 10 years. All-cause subgroup analysis showed heterogeneity between the 2 subgroups in the United States and the United Kingdom. The heterogeneity may be due to different sample sizes, different follow-up times, and different doses of aspirin.

Sensitivity analysis found that the research of Fraser et al^[11]^ was the heterogeneity source of specific death. After rejection, the pooled HR was 0.85, and the 95% confidence interval was 0.76 to 0.96. The sensitivity analysis of all-cause death found that studies^[11,23]^ were outliers. However, after one-by-one rejection, the heterogeneity did not change significantly, suggesting that the meta-analysis results were stable. The reasons for the heterogeneity in this study may be: the difference in sample size, the sample size of Shiao, Blair et al^[16,22]^ was less than 1000, and the sample size of Barron, Mc Menamin et al^[23]^ were greater than 4000. The follow-up time is different, the shortest was 5 years and the longest was 16 years. The dose of aspirin used by the participants is different. It has been reported that different doses of aspirin use different breast cancer survival rates.^[33]^ Sensitivity analysis of recurrence/metastasis was a source of heterogeneity in the future. The reason for the heterogeneity was that the total sample size of Shiao et al^[22]^ was only 222, far lower than other studies, perhaps because of the low quality of Shiao et al.^[22]^

Like other studies, this meta-analysis is also inadequate. The number of studies included was small, no case-control studies were selected, and no statistically selected RR/OR and 95% confidence interval studies were selected. The included studies only compare whether or not to use aspirin, and ignore the influencing factors such as the dose, frequency and course of treatment of aspirin. There are 2 papers with low quality and high risk of bias.

## 5 Conclusion

Overall, this meta-analysis showed that aspirin use may reduce the risk of specific death from breast cancer, all-cause mortality, and recurrence/metastasis.

## Author contributions

**Methodology:** Jiamin Liu.

**Software:** Meng Yang.

**Writing – original draft:** Fengxian Zheng, Xiaoyong Wu.

**Writing – review & editing:** Aimin Liu.
